# Eugenol inhibits preadipocyte differentiation and lipid accumulation via upregulating ATF3

**DOI:** 10.1590/1414-431X2025e14575

**Published:** 2025-10-06

**Authors:** Pei-pei Gu, Lin Xu, Patiguli Kadierjiang, Xin Shen, Jun Liu, Yang Li, Lu Zeng, Hong-mei Lai, Haoshaqiang Zhang, Jing Tao

**Affiliations:** 1 Heart Panvascular Medical Diagnosis and Treatment Center, People's Hospital of Xinjiang Uygur Autonomous Region, Urumqi, Xinjiang, China; 2 Medical Device Center, People's Hospital of Xinjiang Uygur Autonomous Region, Urumqi, Xinjiang, China; 3 Department of Orthopedic Surgery, People's Hospital of Xinjiang Uygur Autonomous Region, Urumqi, Xinjiang, China

**Keywords:** Activating transcription factor 3, Eugenol, Adipocyte differentiation, Adipogenesis, PI3K-AKT pathway

## Abstract

The global prevalence of overweight status and obesity has increased considerably. Obesity is the common pathological basis of numerous diseases and a crucial triggering factor for diabetes. This study aimed to investigate the role of activating transcription factor 3 (ATF3) in the adipogenic process of adipocytes and the related compounds of Chinese medicine potentially targeting ATF3. The differentially expressed genes (DEGs) in high-fat diet (HFD) or overweight patients were identified by analyzing Gene Expression Omnibus (GEO) data profiles GSE112999, GSE112740, and GSE48964. qRT-PCR and western blot were conducted to detect the expression levels of related genes. Oil-red O staining was conducted to detect lipid droplet formation within 3T3-L1 adipocytes. Compounds that interacted with ATF3 were screened through the pharmacological database and analysis platform of the traditional Chinese medicine system. Furthermore, the eugenol effect on ATF3 expression was evaluated. ATF3 expression was increased in the adipose tissues of HFD mice and in clinically obese patients. Knock down of ATF3 promoted adipocyte lipid droplet formation, upregulated the protein levels of adipocyte markers (Fabp4 and PPARγ), elevated intracellular triglyceride (TG) levels, and activated the AKT signaling pathway. Eugenol effectively promoted ATF3 expression and inhibited adipocyte differentiation and adipogenesis. ATF3 expression was found to be elevated within the adipose tissues of overweight patients, whereas ATF3 knockdown promoted adipocyte differentiation and lipid accumulation by activating the PI3K/AKT signaling pathway. Therefore, ATF3 overexpression or supplementation with eugenol may be a potential method for overcoming obesity.

## Introduction

With the substantial improvement in living standards and the changes in food habits in recent years, the number of overweight people worldwide has increased significantly. It is predicted that 57.8% of adults worldwide will suffer from obesity and related issues by 2030, which will pose a significant threat to human health and trigger a heavy societal burden (1). Obesity is a fundamental component in the initiation and progression of multiple metabolic disorders (2,3), but its specific pathogenesis is unclear and accounts for the lack of effective prevention and treatment methods. Therefore, the exploration of obesity mechanisms and the regulatory factors affecting fat accumulation is of prime importance.

Eugenol is a volatile phenolic constituent of clove essential oil, occurring naturally in various aromatic plants such as clove, cinnamon, basil, and nutmeg (4). Eugenol exerts pharmacological effects, including anti-inflammatory, anti-oxidant, anti-viral, and anticancer effects (5). Most experimental studies have targeted the involvement of eugenol in inflammation and oxidative stress (6). However, the pharmacological effects of eugenol on obesity and the potential molecular mechanisms remain unclear.

Activating transcription factor 3 (ATF3), belonging to the ATF/cAMP response element-binding (CREB) family, targets the cyclic AMP response element (CRE) within many promoters having the consensus sequence TGACGTCA, thus regulating gene expression (7). *ATF3* gene encodes 181 amino acids with a calculated molecular mass of 22 kDa (8). *ATF3* plays a crucial role in mediating cellular stress response. *ATF3* mRNA level is low and undetectable in most tissues and cells, but the expression level increases under stress conditions, including chemical toxins, cytokines, and chemokines, being an adaptive response gene (9). As previously evidenced, ATF3 is associated with cellular responses to multiple injuries (such as kidney, heart, and brain ischemia/reperfusion injury, liver injury, and ventilator-induced lung injury) and various types of cell damage (such as damage induced by ultraviolet radiation and doxorubicin) (10). The role of ATF3 in metabolic diseases (such as obesity) has also been investigated. For instance, it has been shown that compared with high-fat diet (HFD)-fed wild-type mice, mice with ATF3 knockout develop metabolic disorders, such as obesity and insulin resistance; ATF3 overexpression can induce the trans-differentiation of white fat to brown fat within HFD mice (11). The above studies suggest that ATF3 may exert a pivotal effect on obesity metabolism. This study aimed to investigate the role of ATF3 in the adipogenic process of adipocytes and the related compounds of Chinese medicine potentially targeting ATF3.

## Material and Methods

### GEO data analysis

Datasets GSE112999, GSE112740, and GSE48964 were downloaded from the Gene Expression Omnibus (GEO) database (https://www.ncbi.nlm.nih.gov/geo/). The GSE112999 dataset was analyzed on white adipose tissues of 6-10 week-old male C57BL/6J mice, including 9 cases with a low-fat diet (LFD) and 9 cases with a high-fat diet (HFD). The GSE112740 dataset was analyzed on brown adipose tissues of 6-10 week-old male C57BL/6J mice, including 3 LFD cases and 3 HFD cases (12). The GSE48964 dataset was analyzed on subcutaneous white adipose tissues obtained from weight loss or liposuction surgery, including 3 cases with a body mass index (BMI)>40 kg/m^2^ and 3 control cases with a BMI<25 kg/m^2^ (13). The R language limma package was used to perform differential gene analysis on these datasets, with the screening standard as |logFC |>1 and corrected by P<0.05. The volcano plot, heat map, and gene expression profiles were plotted using the R language ggplot2 package.

### Subcutaneous adipose tissue preparation

This study included 12 non-diabetic adults that were divided into 6 lean healthy controls (3 males and 3 females, aged 32-44 years, BMI<25 kg/m^2^) and 6 obese individuals (3 males and 3 females, aged 26-48 years, BMI>30 kg/m^2^). These individuals had no serious diseases such as lung, heart, kidney, liver, and blood system diseases, and no childbearing, immune disorder, type 1 and 2 diabetes, or malignant tumors. The abdominal subcutaneous adipose tissues were harvested during bariatric surgery in obese patients or during hiatal hernia surgery in both lean healthy controls and obese patients. All participants gave written informed consent, and the study procedures were approved by the Ethical Committee of Xinjiang Uygur Autonomous Region People's Hospital. The harvested 50-100 mg adipose tissues were washed in cool saline and then stored at -80°C until subsequent analyses.

### Cell culture

The mouse preadipocyte 3T3-L1 (#GNM25) cell line was procured from the Cell Bank of the Chinese Academy of Sciences (China). 3T3-L1 cells were cultivated in DMEM high glucose media (#11965092, Gibco, USA) containing 10% fetal bovine serum (FBS) in an incubator (37°C, 5% CO_2_) with 100% humidity. After growing to 70-80% confluence, cells were trypsinized using 0.25% trypsin to make a single-cell suspension, which was then passaged at a 1:2-1:3 ratio. The cells were cultured for a total of six days to induce adipogenic differentiation. Briefly, 3T3-L1 cells were first cultivated in a complete culture media added with IBMX (0.5 mM), dexamethasone (1 μmol/L), and insulin (3 μg/mL) for 2 days, then in a culture medium containing insulin only for 2 days, and finally in a conventional complete culture medium for 2 days.

### Cell transfection and treatment

si-RNA (si-ATF3, #1, #2, #3) and its negative control (si-NC) (100 nM) were supplied by Guangzhou RiboBio Co., Ltd. (China). Cells were grown to 70-80% confluence in six-well plates. Next, Lipofectamine 2000 reagent (Invitrogen, USA) was used as per the protocol to transfect cells for 24 h with si-RNAs and si-NC. Cells were then induced for adipogenic differentiation, followed by function experiments and extraction of cell RNA and total protein. Cells were divided into groups based on their respective transfected siRNA (Table 1). For eugenol treatment, 3T3-L1 cells were cultured using 10 μM eugenol and then using an induced differentiation medium for 6 days.

**Table 1:** Sequence of siRNAs and qRT-PCR primers.

Name	Gene accession number	Forward primer (5'-3')	Reverse primer (5'-3')
si-ATF3 #1 mouse	NM_007498	CAGAAUAAACACCUCUGCCTT	GGCAGAGGUGUUUAUUCUGTT
si-ATF3 #2 mouse	NM_007498	UGCUGCCAAGUGUCGAAACTT	GUUUCGACACUUGGCAGCATT
si-ATF3 #3 mouse	NM_007498	AGAAGGAACAUUGCAGAGCTT	GCUCUGCAAUGUUCCUUCUTT
si-NC mouse		UUCUCCGAACGUGUCACGUTT	ACGUGACACGUUCGGAGAATT
qRT-PCR ATF3 human	XM_054336744.1	CCTCTGCGCTGGAATCAGTC	TTCTTTCTCGTCGCCTCTTTTT
qRT-PCR β-actin human	NM_001101.5	CACACAGGGGAGGTGATAGC	GACCAAAAGCCTTCATACATCTCA
qRT-PCR FOS human	NM_005252.4	CTTCACCCTGCCTCTCCTCAA	GCTGGGAACAGGAAGTCATCAA
qRT-PCR CCL2 human	NM_002982.4	CAGCCAGATGCAATCAATGCC	TGGAATCCTGAACCCACTTCT
qRT-PCR ATF3 mouse	NM_007498.3	CGCTGGAGTCAGTTACCGTC	ATTTTATTTCTTTCTCGCCGC
qRT-PCR β-actin mouse	NM_007393.5	GGCTGTATTCCCCTCCATCG	CCAGTTGGTAACAATGCCATGT

### Quantitative real-time polymerase chain reaction (qRT-PCR)

Trizol (Invitrogen) was used to extract total RNA from cells and tissues. A reverse transcription kit (TaKaRa, Japan) was used following the instructions to reverse transcribe total RNA. LightCycler 480 fluorescence quantitative PCR instrument (Roche Diagnostics, USA) was used to detect gene expression. The reaction conditions followed the operating protocol of the fluorescence quantitative PCR kit (SYBR Green Mix, Roche Diagnostics). The PCR cycling temperature conditions were as follows: initial denaturation at 95°C for 10 s, 40 cycles of denaturation at 95°C for 5 s, annealing at 60°C for 10 s, and elongation at 72°C for 10 s. The final cycle was followed by an extension at 72°C for 5 min. Each quantitative PCR reaction was carried out three times. β-actin was used for the normalization of the relative expression of target genes, which was calculated using the 2^-ΔΔCt^ method: ΔΔCt = experimental group (Ct target gene - Ct internal reference) - control group (Ct target gene - Ct internal reference). The primers and gene sequences are shown in Table 1.

### Western blot

RIPA lysis buffer (Beyotime, China) was used to lyse cells, thereby yielding whole-cell protein samples. A membrane protein extraction kit (C500049, Sangon Biotech Co., Ltd., China) was also used to extract membrane proteins. The BCA reagent kit (Beyotime) was used to determine the protein content. Subsequently, after adding protein samples and mixing with the sample buffer (Beyotime), a 5-min boiling water bath was used to heat the mixture to induce protein denaturation. The electrophoresis of the proteins was carried out using an initial constant voltage of 80 V for 30 min until the bromophenol blue indicator reached the bottom edge, after which the voltage was switched to 120 V for 1-2 h, and electrophoresis was stopped. The transfer was carried out in an ice bath with a transfer current of 220 mA for 120 min. Subsequently, after 1-2 min-washing with the washing solution, membranes were sealed at room temperature (RT) for 60 min in the sealing solution, followed by overnight incubation at 4°C using primary antibodies: α-tubulin (#77763, 1:1000, Cell Signaling Technology (CST), USA), ATF3 (#18665, 1:1000, CST), Fabp4 (#50699, 1:1000, CST), PPARγ (#2443, 1:1000, CST), p-AKT (Ser473; # 4060, 1:1000, CST), and AKT (#4685, 1:1000, CST). On the next day, membranes were rinsed three times for 10 min each, followed by a 1-h incubation at RT using horseradish peroxidase (HRP)-conjugated secondary antibody goat anti-rabbit or anti-mouse IgG (1:5000, Protientech, China). The membranes were subsequently rinsed three times for 10 min per rinse. A chemiluminescence imaging system (Tannon-5200, Tannon, China) was used to develop and visualize membranes.

### Oil red O staining

Cells were plated (2×10^5^ cells/well) onto 24-well plates, with 3 duplicated wells per group. After reaching 60-70% confluence, the treatment was carried out. The culture media was removed after treatment. Cells were subsequently rinsed three times using pre-cooled PBS, followed by a 30-min incubation at RT using 300 μL 4% paraformaldehyde. Cells were washed three times in PBS, and then 300 μL of Oil red O working solution was added for 30-min staining at RT. Next, cells were rinsed three times in PBS, and an inverted microscope (Olympus, Japan) was used to observe and photograph cells.

### Colorimetric assay detecting intracellular triglyceride (TG) content

The TG content detection kit (A110-1-1, Nanjing JianCheng Bioengineering Institute, China) was used to measure the intracellular TG content. Cells (5×10^6^ cells) were collected before removing the supernatant. One milliliter of extraction buffer was added to cells and thoroughly mixed. After ultrasonic crushing for 1 min, cells were subjected to 10-min centrifugation at 8000 *g* at 4°C prior to collecting the supernatant. Next, the protein concentration of supernatant samples was determined by the BCA Protein Assay kit (Beyotime). The blank, standard, and test tubes were prepared per the reagent kit's protocol. Samples (2.5 μL) and 250 μL working solution were subsequently plated onto 96-well plates for 10 min at 37°C, and the absorbance at 510 nm of each tube sample (blank, standard, and test) was measured. TG content (mmol/gprot) = (absorbance test - absorbance blank) ÷ (absorbance standard - absorbance blank) × standard concentration (mmol/L) ÷ cell protein concentration (gprot/L).

### Cell Counting Kit-8 (CCK-8) assay

The CCK-8 assay (#C0038, Beyotime) was used to assess cell viability as directed by the manufacturer. 3T3-L1 cells were treated as mentioned above, each well was supplemented with CCK-8 reagent, and a microplate reader was applied to measure absorbance at 450 nm wavelength (14). The absorbance values across different groups were compared to evaluate cell viability.

### Statistical analysis

The experimental data are reported as means±SD. GraphPad Prism 8.0 software was used to perform statistical analyses and generate diagrams. The unpaired Student's *t*-test was used to analyze the differences between the 2 groups. One-way ANOVA followed the Tukey *post hoc* test was applied to analyze more than 2 groups. The significance level was set at P<0.05.

## Results

### ATF3 expression was upregulated in obesity-related adipose tissues

**Figure 1:**
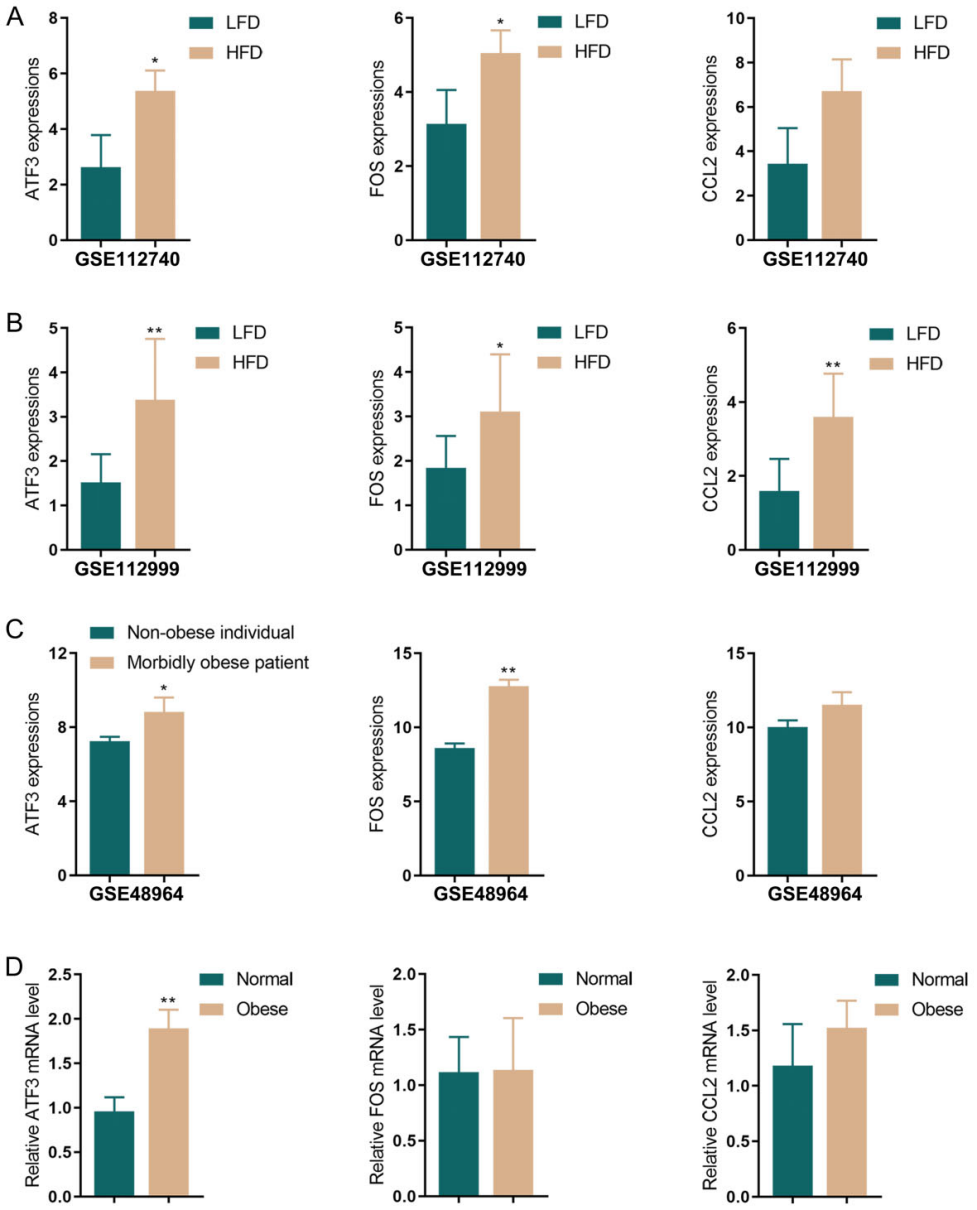
ATF3 expression was upregulated in obesity-associated adipose tissues. ATF3, FOS, and CCL2 mRNA levels in dataset (**A**) GSE112740 (n=3), (**B**) GSE112999 (n=9), and (**C**) GSE489649 (n=3) were detected. **D**, qRT-PCR was used to detect ATF3, FOS, and CCL2 mRNA levels in clinical samples. Data are reported as means and SD; n=6 (biological replicates). *P<0.05, **P<0.01; Student's *t*-test. LFD: low-fat diet; HFD: high-fat diet.

Compared to the normal control, there were 262 upregulated and 203 downregulated genes in dataset GSE112999 (Supplementary Figure S1A), and 295 upregulated and 344 downregulated genes in dataset GSE112740 (Supplementary Figure S1B) within the white adipose tissue of the HFD-fed group. The intersection of the two datasets yielded 41 co-upregulated genes and 17 co-downregulated genes with significant differential expression (Supplementary Figure S1C). Moreover, within the GSE48964 chip containing human adipose tissues, compared with the non-obese individuals (BMI<25 kg/m^2^), there were 100 upregulated and 53 downregulated genes (Supplementary Figure S1D) in cases with BMI>40 kg/m^2^. Next, these three datasets were intersected, and then three common DEGs were obtained: *ATF3*, *FOS*, and *CCL2*. These three genes were upregulated in datasets GSE112999, GSE112740, and GSE48964 (Figure 1A-C). Next, the expression of these three genes was validated in clinical subcutaneous adipose tissues. As indicated by the results, ATF3 mRNA expression was noticeably increased in the adipose tissues of the obese mice compared to the normal control mice (Figure 1D). Therefore, ATF3 was selected as the research object for subsequent experiments.

### ATF3 knockdown promoted adipocyte lipogenesis

**Figure 2:**
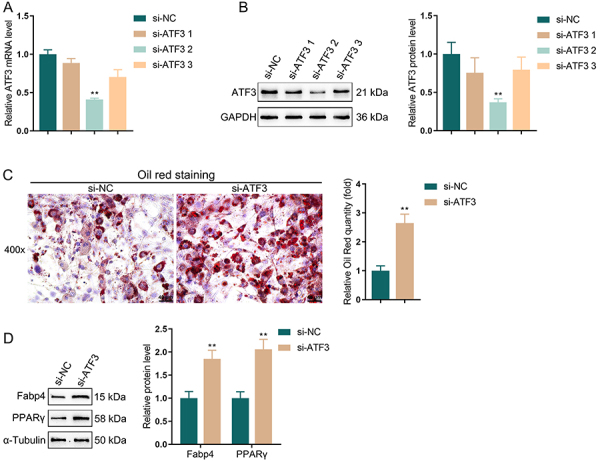
ATF3 knockdown promoted adipocyte lipogenesis. The knockdown efficiency of si-ATF3 was detected using qRT-PCR (**A**) and western blot (**B**). After induction of lipid formation, Oil red O staining was applied to detect the formation of lipid droplets in cells (**C**); magnification 400×, scale bar 40 μm. **D**, Western blot was conducted to detect the protein level of adipocyte markers (Fabp4 and PPARγ). Data are reported as means and SD; n=3 (biological replicates). **P<0.01, *vs* the si-NC (negative control) group; ANOVA and Student's *t*-test.

According to the GSE expression profiles and the results of clinical samples, ATF3 was notably upregulated in HFD-induced mice and obesity-related adipose tissues. Therefore, ATF3 may be involved in adipocyte adipogenesis. For validation, 3T3-L1 cells were cultured and induced for adipogenic differentiation (Supplementary Figure S2). Compared to the normal group, 3T3-L1 cells showed a marked increase in both lipid droplet accumulation (Supplementary Figure S2A) and the expression levels of Fabp4 and PPARγ (Supplementary Figure S2B) following differentiation. These results indicated that the 3T3-L1 cells were effectively differentiated into mature adipocytes. Herein, ATF3 expression was knocked down with siRNAs, and si-ATF3-2 showed the best knockdown efficiency (Figure 2A and B), referred to as si-ATF3. 3T3-L1 cells were transfected with si-ATF3, and then induced for adipogenic differentiation. si-ATF3-transfected cells showed significantly increased lipid droplet formation compared to si-NC cells (Figure 2C). Additionally, ATF3 effects on the protein level of adipocyte markers (Fabp4 and PPARγ) were evaluated. The results showed that ATF3 knockdown remarkably elevated the protein level of Fabp4 and PPARγ compared to the normal control (Figure 2D). In summary, silencing ATF3 can promote adipogenesis in adipocytes, and ATF3 played a role in inhibiting adipogenic differentiation.

### ATF3 knockdown increased TG content and activated the AKT pathway in adipocytes

**Figure 3:**
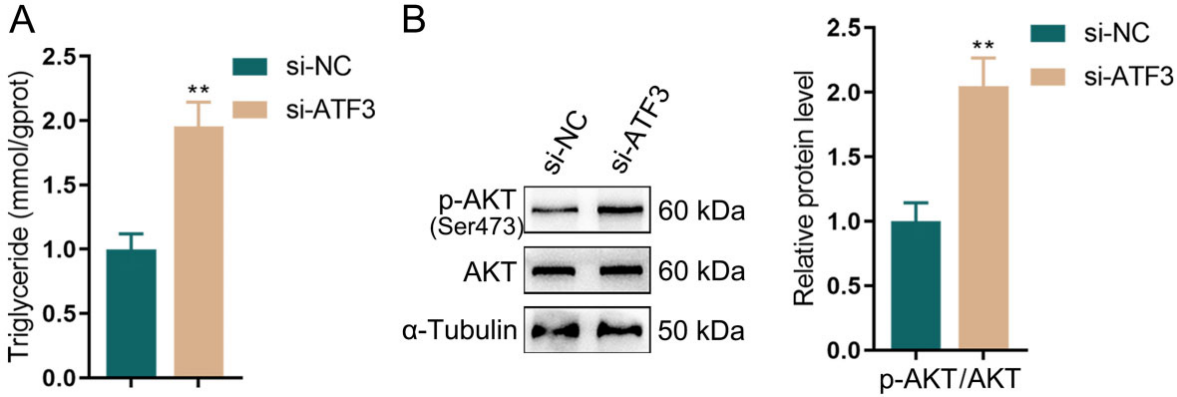
ATF3 knockdown increased triglyceride (TG) content and activated the AKT pathway in adipocytes. After si-ATF3 transfection and 3T3-L1 cell adipogenic differentiation, (**A**) colorimetric assay was employed to detect the intracellular TG content. **B**, Western blot was applied to detect the total AKT and phosphorylated AKT (Ser473 residue) levels. Data are reported as means and SD; n=3 (biological replicates). **P<0.01, compared to the si-NC (negative control) group; ANOVA and Student's *t*-test.

The PI3K-AKT signaling was shown to exert a crucial effect on adipogenic differentiation and adipogenesis (15,16). According to the results, after si-ATF3 transfection and then adipogenic differentiation, 3T3-L1 cells had notably higher TG content in adipocytes than the control cells (Figure 3A). Moreover, the phosphorylated level of AKT was noticeably elevated (Figure 3B). Based on the above, ATF3 knockdown may promote adipocyte differentiation and lipid formation by activating the PI3K-AKT signaling pathway.

### The target gene of ATF3 was screened using the pharmacological network

**Figure 4:**
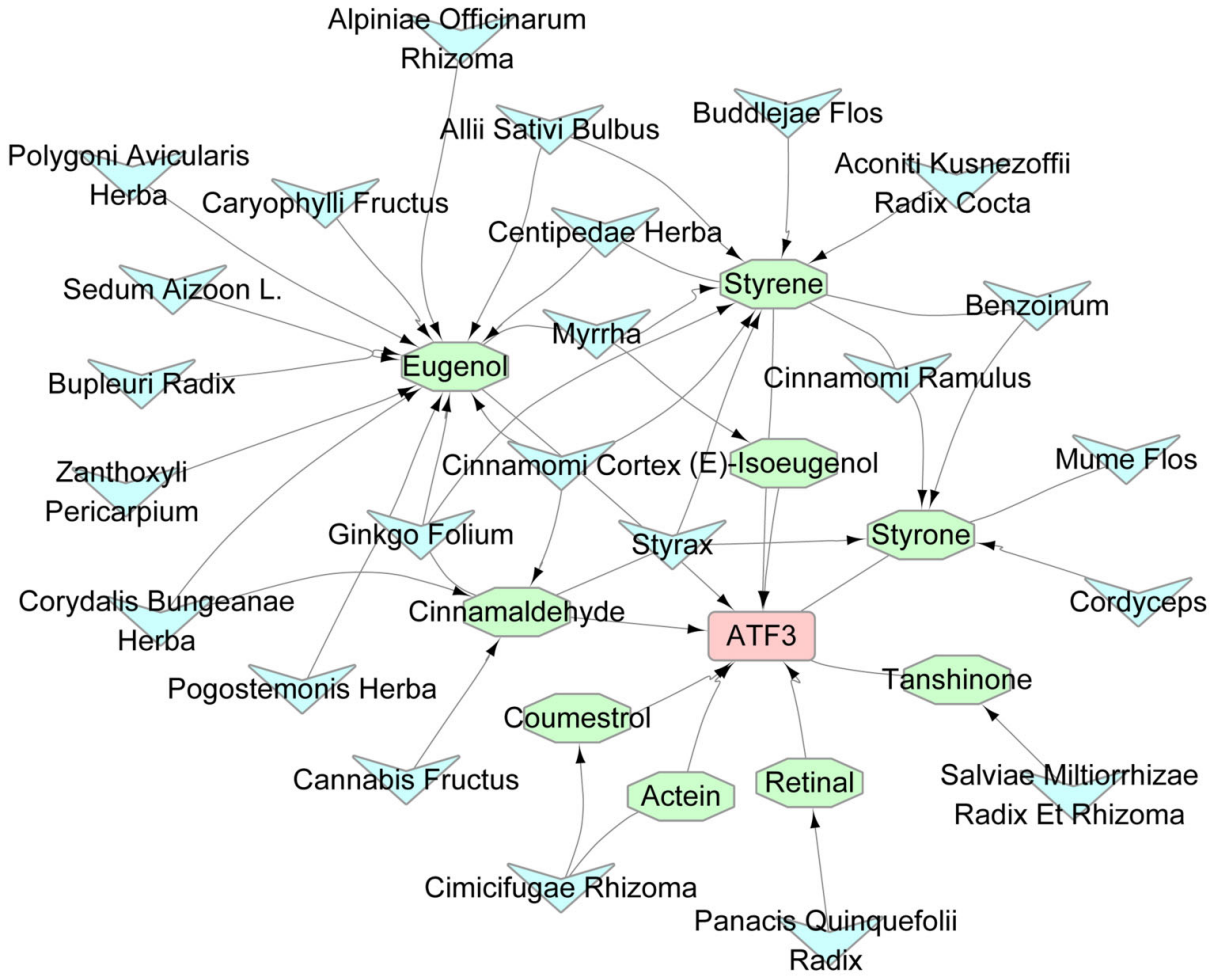
The ATF3 target gene was screened using the pharmacological network. Herbs and their compounds that can interact with ATF3 target genes were screened using traditional Chinese medicine system pharmacology database and analysis platform.

The herbs and their ingredients potentially interacting with ATF3 target genes were screened from the traditional Chinese medicine system pharmacology database and analysis platform (TCMSP; https://tcmsp-e.com/). A total of 9 active compounds (retinal, styrene, cinnamaldehyde, coumestrol, styrone, (E)-isoeugenol, tanshinone, actein, and eugenol) (OB>20) that can bind to ATF3 were obtained (Figure 4). Among these active compounds, eugenol, styrene, and cinnamaldehyde were the main compounds. As indicated by the results, most active compounds acted on ATF3 through the mediation of eugenol; hence, eugenol was selected as the subsequent research object.

### Eugenol inhibited adipogenic differentiation and lipid formation

**Figure 5:**
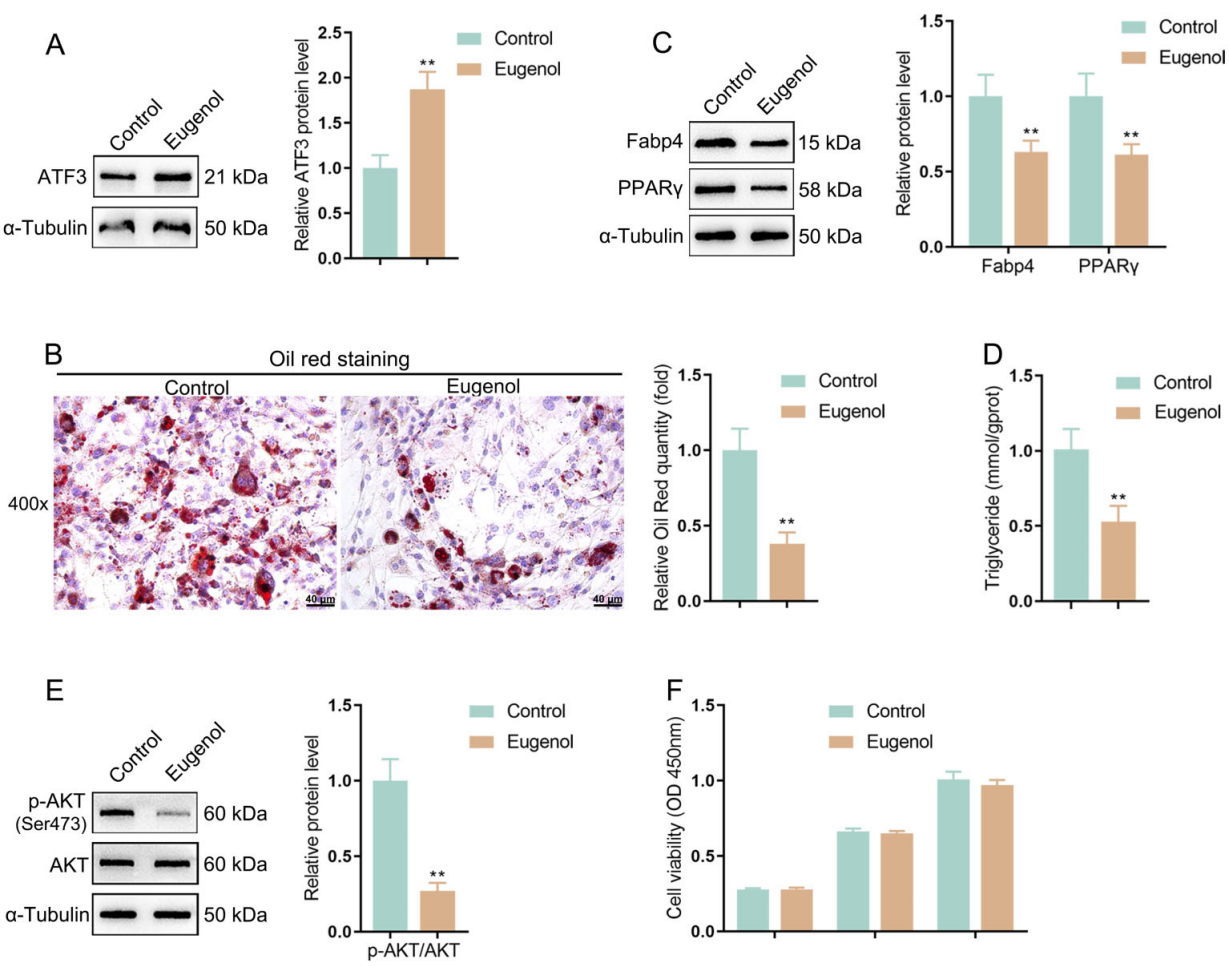
Eugenol inhibited adipogenic differentiation and lipid formation. **A**, In adipocytes with adipogenic induction, a Western blot was applied to detect the regulatory effect of eugenol on the ATF3 protein level. **P<0.01, *vs* normal control. **B**, Oil red O staining was employed to evaluate the lipid droplet formation in cells; magnification 400×; scale bar 40 μm. **C**, Western blot was carried out to detect the protein content of adipocyte markers (Fabp4 and PPARγ). **D**, Colorimetric assay was conducted to detect the intracellular triglyceride level. **E**, Western blot was performed to detect total AKT and phosphorylated AKT (Ser473 residue) levels within adipocytes. **F**, CCK-8 assay was used to detect cell viability. Data are reported as means and SD; n=3 (biological replicates). **P<0.01 *vs* the control group; ANOVA and Student's *t*-test.

Eugenol effects on adipocytes after adipogenic induction were evaluated. It was found that eugenol notably elevated ATF3 protein levels in adipocytes after adipogenic induction (Figure 5A). Additionally, eugenol effectively decreased the lipid droplet formation within adipocytes (Figure 5B), inhibited the protein levels of adipocyte markers (Fabp4 and PPARγ) (Figure 5C), and reduced intracellular TG contents (Figure 5D). Moreover, eugenol also remarkably inhibited the phosphorylated level of AKT (Figure 5E). However, eugenol did not significantly affect cell viability of adipocytes (Figure 5F). Taken together, eugenol inhibited adipocyte maturation and differentiation as well as lipid formation, which might be mediated by the inhibition of the PI3K-AKT signaling.

## Discussion

Obesity has become the main risk factor affecting global human health in the 21st century (17). According to WHO statistics, approximately 650 million adults and 43 million children under the age of 5 are overweight (BMI≥30) (18). China ranked highest in the number of obese people worldwide (19). A series of obesity-induced metabolic disorders have shown a rapid growth trend, including type 2 diabetes, non-alcoholic fatty liver disease, and cardiovascular and cerebrovascular disorders (20,21). It has been confirmed that excessive lipid accumulation in white adipose tissues is strongly associated with the proliferation and hypertrophy of adipocytes (22). Therefore, regulating adipocyte differentiation and intracellular TG content may help prevent and treat metabolic diseases (such as obesity). Herein, ATF3 knockdown has been shown to promote pre-adipocyte adipogenic differentiation and exacerbated TG accumulation, indicating that ATF3 may contribute to inhibiting fat formation within obesity initiation and progression.

An increasing number of studies have proposed that ATF3 acts as a risk factor for metabolic disorders, including obesity (23,24). However, the protective role of ATF3 in animal models of metabolic diseases has also been revealed (25,26). Currently, there is no consensus on ATF3 effects (harmful or beneficial) on metabolic processes, which may depend on different tissues, organs, and stress signals (27). In this study, ATF3 expression level within the adipose tissue of HFD mice and obese individuals was found to be upregulated compared to that in LFD mice and normal BMI individuals through retrieval of GSE datasets from the GEO database. Meanwhile, clinical adipose tissue samples were collected, and obese patients showed elevated ATF3 expression levels in adipose tissues relative to individuals with a normal BMI. However, knocking down ATF3 could promote adipogenic differentiation and lipid droplet formation. PPARγ is known as a specific transcription factor that can regulate adipocyte differentiation by modulating C/EBPα and Fabp4 expressions (28). Fabp4 is a potent marker for the adipocyte differentiation ability of mature adipocyte cytoplasmic proteins (29). Similarly, herein, ATF3 knockdown could promote the expression levels of lipid formation markers (Fabp4 and PPARγ), suggesting that ATF3 expression level might be protectively increased within obesity. Aligned with previous studies, ATF3 has been revealed to inhibit adipogenic differentiation and PPAR γ expression levels within 3T3-L1 cells (30,31).

Subsequently, the underlying mechanism was investigated. It has been shown that the phosphorylated AKT in the PI3K-AKT pathway can penetrate the nucleus, which inhibits foxo1 phosphorylation and thereby relieves the inhibition of PPARγ activity, thereby promoting lipid formation (32). Additionally, as previously reported, the PI3K-AKT signaling was tightly associated with lipid synthesis; suppressing PI3K-AKT signaling activation could block insulin digestion and inhibit glucose uptake and fat synthesis in cells. Herein, ATF3 has been proven to regulate the AKT pathway activation in adipocytes and inhibit TG production. Eugenol has been used for dietary intervention to treat metabolic diseases such as diabetes and obesity. Eugenol is the main component of cinnamon and possesses antibacterial and anti-inflammatory activities. It is also involved in regulating metabolic problems (33). A previous study has indicated that eugenol may regulate oxidative stress and gut microbiota to improve HFD-induced lipid metabolism disorder (34). The underlying mechanism of eugenol in regulating obesity was further investigated in this study. It was found that ATF3 may be a target gene of eugenol through the public database TCMSP. After a series of further experimental results, eugenol was evidenced to upregulate ATF3 expression, inhibit adipogenic differentiation, and reduce TG content. These results provided a reference for the molecular mechanism of eugenol alleviating obesity.

In conclusion, this study demonstrated for the first time that eugenol could inhibit adipogenic differentiation and lipid accumulation by upregulating ATF3 expression levels and inhibiting the PI3K/AKT signaling pathway during adipogenic differentiation. Hence, eugenol supplementation or ATF3 overexpression may be a promising method for overcoming obesity.

## Supplementary Material

Click here to view [pdf].
