# Supplementary material for: Eugenol inhibits preadipocyte differentiation and lipid accumulation via upregulating ATF3
Source: Braz J Med Biol Res. 2025 Oct 6;58:e14575. doi: 10.1590/1414-431X2025e14575 (PMC12513698; doi:10.1590/1414-431X2025e14575)

Figure S1. The obesity-induced differentially expressed genes (DEGs) in adipose tissues were screened. **A**, Adipose tissues in dataset GSE112740 (containing 3 high-fat diet (HFD) and 3 low-fat diet (LFD) mice) were analyzed, and 295 increased genes (log2FC>1, $P<0.05$) and 344 decreased genes (log2FC<–1, $P<0.05$) were obtained. **B**, The dataset GSE112999 (containing 9 HFD and 9 LFD mouse cases) was analyzed using the limma tool. The DEGs in the adipose tissues of HFD and LFD mice were screened, and 262 increased genes (log2FC>1, $P<0.05$) and 203 decreased genes (log2FC<–1, $P<0.05$) were finally obtained. **C**, DEGs in datasets GSE112999 and GSE112740 were intersected, and 41 common upregulated genes (log2FC>1, $P<0.05$) and 17 common decreased genes (log2FC<–1, $P<0.05$) were identified. **D**, The subcutaneous adipose tissues of 3 morbidly obese patients and 3 lean individuals in GSE48964 were analyzed, and 100 increased genes (log2FC>1, $P<0.05$) and 53 decreased genes (log2FC<–1, $P<0.05$) were obtained.

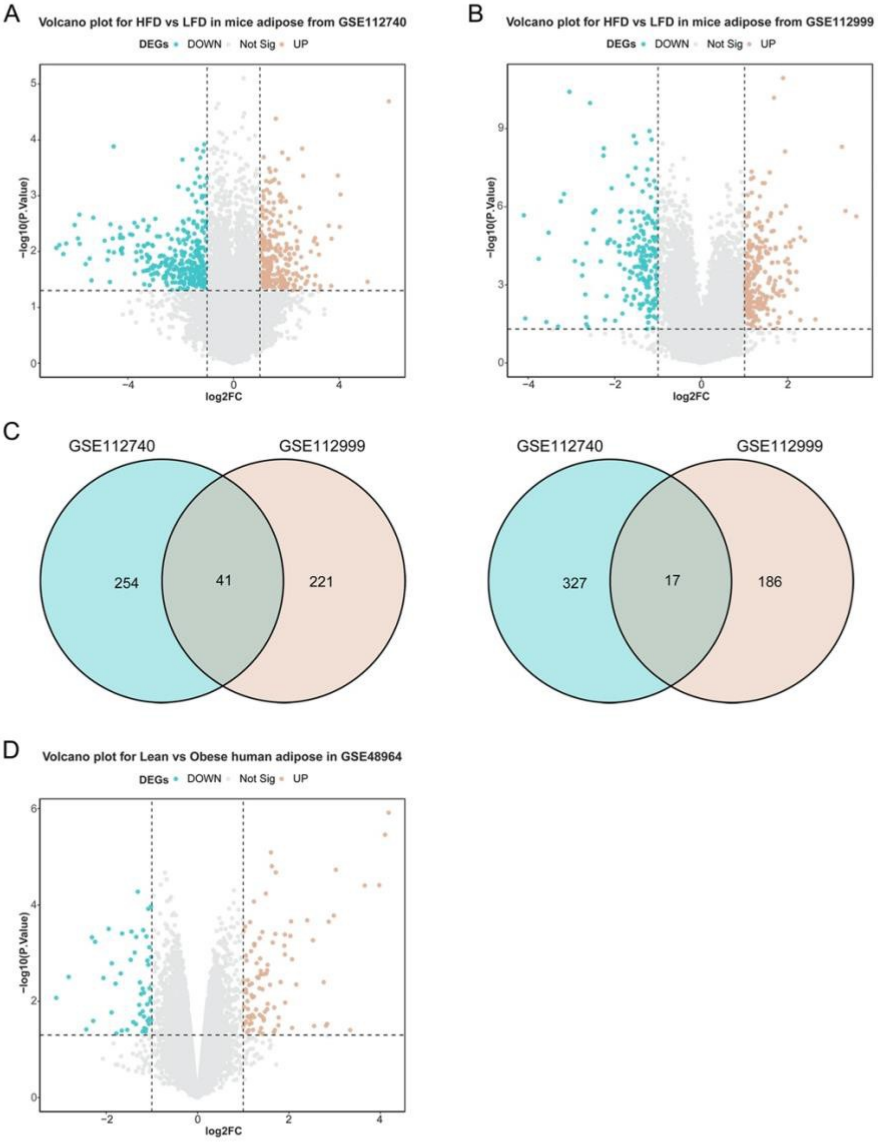

Figure S2. Validation of the adipogenic differentiation cell model. 3T3-L1 cells were cultured for a total of six days to induce adipogenic differentiation. **A**, After induction of lipid formation, Oil red O staining was used to detect the formation of lipid droplets in cells; magnification 400×, scale bar 40 μm. **B**, Western blot was used to detect the protein level of adipocyte markers (Fabp4 and PPARγ). Data are reported as means and SD; n=3 (biological replicates). **P<0.01 *vs* the normal group; Student's *t*-test.

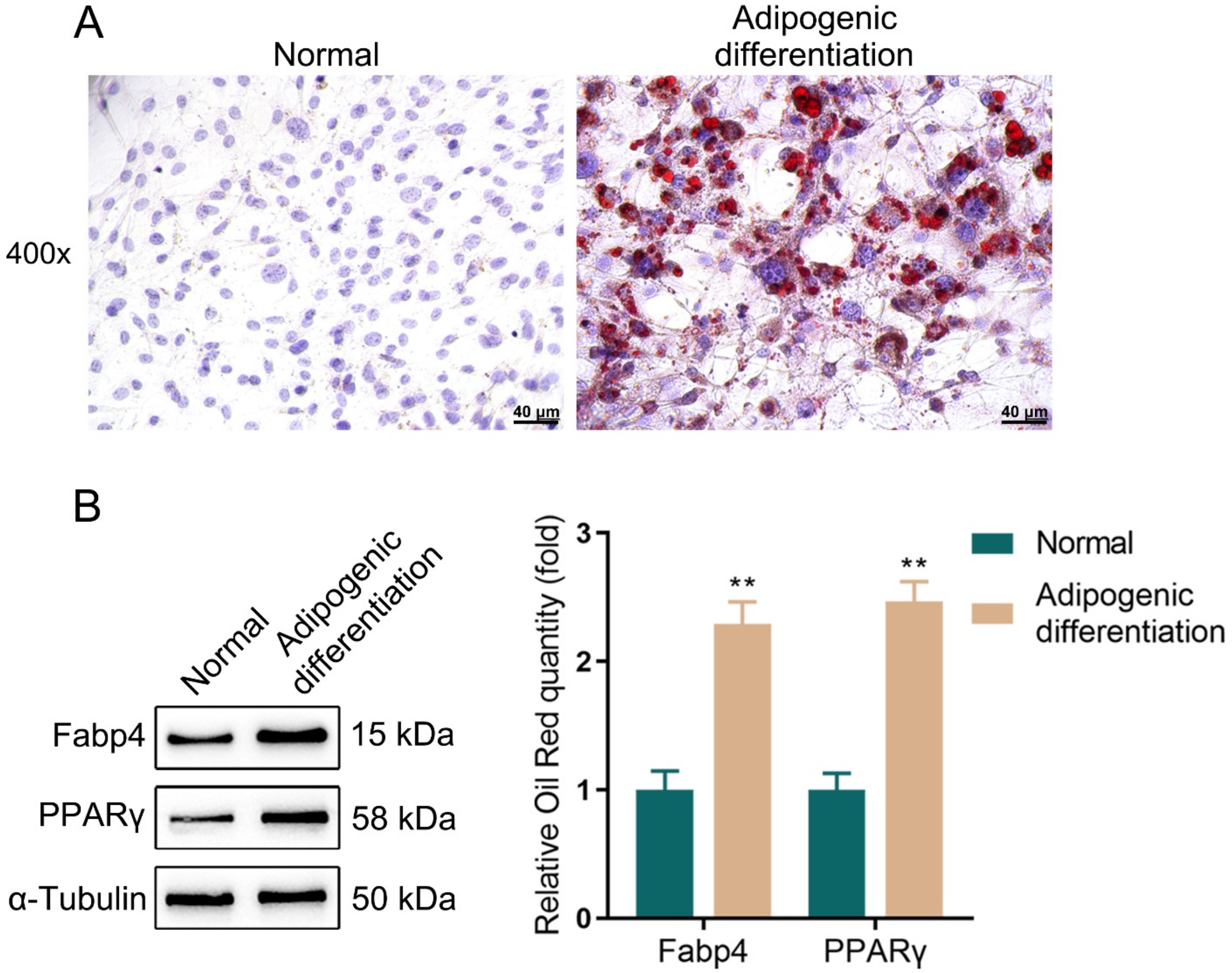

Supplement: Supplementary file 1 [file 1414-431X-bjmbr-58-e14575-suppl.pdf]
